# A Semi-Analytical Extraction Method for Interface and Bulk Density of States in Metal Oxide Thin-Film Transistors

**DOI:** 10.3390/ma11030416

**Published:** 2018-03-11

**Authors:** Weifeng Chen, Weijing Wu, Lei Zhou, Miao Xu, Lei Wang, Honglong Ning, Junbiao Peng

**Affiliations:** 1 State Key Laboratory of Luminescent Materials and Devices, South China University of Technology, Guangzhou 510640, China; chenweifengchn@foxmail.com (W.C.); xumiao4049@126.com (M.X.); mslwang@scut.edu.cn (L.W.); ninghl@scut.edu.cn (H.N.); psjbpeng@scut.edu.cn (J.P.); 2 Guangzhou New Vision Opto-Electronic Technology Co., Ltd., Guangzhou 510530, China; zhoulei@newvision-cn.com

**Keywords:** capacitance–voltage (C–V) characteristics, current–voltage (I–V) characteristics, density of states (DOS), metal oxide thin-film transistors (TFTs)

## Abstract

A semi-analytical extraction method of interface and bulk density of states (DOS) is proposed by using the low-frequency capacitance–voltage characteristics and current–voltage characteristics of indium zinc oxide thin-film transistors (IZO TFTs). In this work, an exponential potential distribution along the depth direction of the active layer is assumed and confirmed by numerical solution of Poisson’s equation followed by device simulation. The interface DOS is obtained as a superposition of constant deep states and exponential tail states. Moreover, it is shown that the bulk DOS may be represented by the superposition of exponential deep states and exponential tail states. The extracted values of bulk DOS and interface DOS are further verified by comparing the measured transfer and output characteristics of IZO TFTs with the simulation results by a 2D device simulator ATLAS (Silvaco). As a result, the proposed extraction method may be useful for diagnosing and characterising metal oxide TFTs since it is fast to extract interface and bulk density of states (DOS) simultaneously.

## 1. Introduction

Thin film transistors (TFTs) are one kind of field-effect transistors (FETs). Their structure and operation principles are similar to those of metal oxide semiconductor field effect transistors (MOSFETs), which are commonly used in modern integrated circuits (ICs) [1]. Amorphous oxide semiconductor thin-film transistors (AOS TFTs) are considered one of the most promising technologies for flat panel display (FPD) due to transparency, good uniformity, higher mobility than hydrogenated amorphous silicon TFTs (a-Si:H TFTs), good process compatibility with a-Si TFTs, and a lower temperature fabrication process than low temperature poly-silicon (LTPS) TFTs [2,3,4,5]. The electrical characteristics of AOS TFTs are significantly affected by the bulk density of states (DOS) in the active layer and the interface density of states between the gate insulator and the active layer. The bulk DOS may be attributed to structural disorder (bond angles and length variations), dangling bonds, non-stoichiometry, and carrier scattering in the amorphous films [6]. The interface DOS may be caused by defects located at the interface, which can exchange charge with the active layers by capturing or emitting electrons [7]. It is important to extract DOS of TFTs for device design, process characterization, or modeling. Several methods have been developed to extract only the bulk DOS, such as numerical calculations [8], optical illumination [9], temperature-dependent characteristics [10], or multi-frequency current–voltage (C–V) characteristics [11]. However, it is usually difficult to extract the interface DOS and the bulk DOS simultaneously. 

Lui et al. [12] and Kimura [13] extracted the interface and bulk density of states in TFTs from the characteristics of capacitance–voltage and current–voltage based on the numerical iterative solution of Poisson’s equation. However, the numerical iterative solution generally has the disadvantages of operation complexity, long computational time, and possible convergence problems. Hastas et al. extracted interface trap states from analysis of the transfer characteristics in the sub-threshold region and the bulk trap states by numerically fitting the surface potential equation [14]. Tsuji et al. extracted the interface trap density based on an expression of interface trap charge density (*Q_it_*) as functions of the front and back-side surface potentials, in which the electric field at the back side is assumed to be zero [15]. Therefore, it is essential to develop analytical methods for extracting both the interface DOS and the bulk DOS of TFTs.

In this work, we propose a semi-analytical method to extract the interface DOS and the bulk DOS in indium zinc oxide (IZO) TFTs simultaneously by only using the low-frequency capacitance–voltage characteristics and current–voltage characteristics.

## 2. Extraction Method

**Figure 1:**
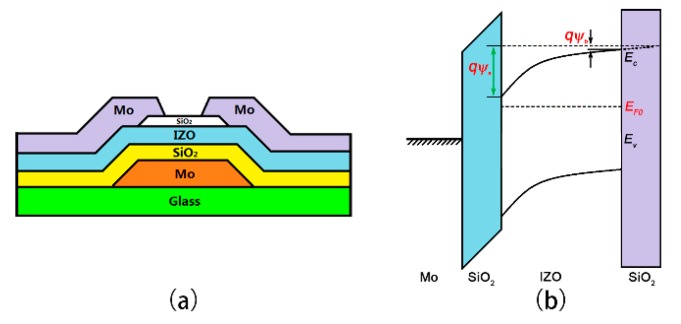
(**a**) Cross-sectional view of indium zinc oxide thin-film transistors (IZO TFTs) with inverted staggered bottom gate structure; (**b**) energy band diagram along the thin-film depth direction of IZO TFTs.

Figure 1a shows a schematic cross-sectional view of indium zinc oxide (IZO) TFTs with inverted staggered bottom gate structure. The IZO material as the channel layer has the advantages of transparency, high mobility, and excellent drain current saturation [16]. Figure 1b shows the energy band diagram of the device, in which the surface potential (*Ψ_s_*) and the back interface potential (*Ψ_b_*) are respectively labeled. Poisson’s equation is given by

(1) $$\frac{d^{2}\psi }{dx^{2}}=-\frac{\rho \left(x\right)}{\varepsilon_{s}}=\frac{q}{\varepsilon_{s}}\left[n_{free}\left(x\right)+n_{trap}\left(x\right)\right]$$

where *n_free_(x)* and *n_trap_(x)* are the free electron concentration and localised trapped electron concentration, respectively.

Using the relationship $\frac{d^{2}\psi }{dx^{2}}=\frac{1}{2}\frac{d}{d\psi }{\left(\frac{d\psi }{dx}\right)}^{2}$, we can get

(2) $${{\left(\frac{d\psi }{dx}\right)}^{2}|}_{\psi =\psi_{s}}-{{\left(\frac{d\psi }{dx}\right)}^{2}|}_{\psi =\psi_{b}}=2\int_{\psi_{b}}^{\psi_{s}}\frac{d^{2}\psi }{dx^{2}}d\psi$$

Substituting Equation (1) into Equation (2) and differentiating both sides with respect to *Ψ*, we have

(3) $$\frac{d}{d\psi_{s}}{{\left(\frac{d\psi }{dx}\right)}^{2}|}_{\psi =\psi_{s}}=-2\frac{\rho (\psi_{s})}{\varepsilon_{s}}$$

(4) $$\frac{d}{d\psi_{b}}{{\left(\frac{d\psi }{dx}\right)}^{2}|}_{\psi =\psi_{b}}=-2\frac{\rho (\psi_{b})}{\varepsilon_{s}}$$

where *ρ*(*Ψ_s_*) is the surface charge concentration, and *ρ*(*Ψ_b_*) is the back interface charge concentration. Equations (3) and (4) can be further rearranged as

(5) $$2{\left(\frac{d\psi }{dx}\right)|}_{\psi =\psi_{s}}\frac{d}{d\psi_{s}}{\left(\frac{d\psi }{dx}\right)|}_{\psi =\psi_{s}}=-2\frac{\rho (\psi_{s})}{\varepsilon_{s}}$$

(6) $$2{\left(\frac{d\psi }{dx}\right)|}_{\psi =\psi_{b}}\frac{d}{d\psi_{b}}{\left(\frac{d\psi }{dx}\right)|}_{\psi =\psi_{b}}=-2\frac{\rho (\psi_{b})}{\varepsilon_{s}}$$

Based on Gauss’s theorem, the surface charge per unit area in the channel (*Q_s_*) considering the effect of the back interface potential can be expressed as

(7) $$Q_{s}=\varepsilon_{s}{\frac{d\psi }{dx}|}_{\psi =\psi_{s}}-\varepsilon_{s}{\frac{d\psi }{dx}|}_{\psi =\psi_{b}}$$

In this paper, the potential distribution along the depth direction (*x*) of the active layer is assumed as

(8) $$\psi \left(x\right)=\psi_{s}\exp\left(-\frac{x}{x_{0}}\right)$$

where *x*_0_ is the characteristic length of the potential distribution. Note that the assumption for Equation (8) will be confirmed by the numerical solution of Poisson’s equation in Appendix A and the device simulation in Section 3. Substituting Equation (8) into Equations (5)–(7), we have

(9) $$\rho (\psi_{s})=-\varepsilon_{s}\frac{\psi_{s}}{{x_{0}}^{2}}$$

(10) $$\rho (\psi_{b})=-\varepsilon_{s}\frac{\psi_{b}}{{x_{0}}^{2}}$$

(11) $$Q_{s}=-\varepsilon_{s}\left(\frac{\psi_{s}}{x_{0}}-\frac{\psi_{b}}{x_{0}}\right)=x_{0}\left(\rho (\psi_{s})-\rho (\psi_{b})\right)$$

While in [12], the surface charge at the back interface was not taken into account.

The differential of surface potential with respect to gate-source bias can be expressed as [17]

(12) $$\frac{d\psi_{s}}{dV_{gs}}=1-\frac{dQ_{g}/dV_{gs}}{C_{ox}}=1-\frac{C_{g}(V_{gs})}{C_{ox}}$$

where *C_g_*(*V_gs_*) is gate capacitance at some *V_gs_*, *C_ox_* is the gate oxide capacitance per unit area, and *Q_g_* is the charge per unit area at the gate electrode

(13) $$Q_{g}=\int_{0}^{V_{gs}}C_{g}\left({V_{gs}}^{\prime }\right)d{V_{gs}}^{\prime }$$

The nonlinear relation between *Ψ_s_* and *V_gs_* can be obtained by integrating Equation (12) over *V_gs_*:

(14) $$\psi_{s}=\int_{V_{fb}}^{V_{gs}}\left(1-\frac{C_{g}\left({V_{gs}}^{\prime }\right)}{C_{ox}}\right)d{V_{gs}}^{\prime }$$

where *V_fb_* is the flat band voltage.

The free charge density per unit area (*Q_i_*) can be expressed by an integration of *n_free_*(*x*) over *x*.

(15) $$Q_{i}\left(V_{gs}\right)=-q\int_{0}^{t_{s}}n_{0}\exp\left(\frac{\psi \left(x\right)}{V_{t}}\right)dx=-q\int_{0}^{t_{s}}n_{0}\exp\left(\frac{\psi_{s}\exp\left(-\frac{x}{x_{0}}\right)}{V_{t}}\right)dx$$

where *t*_s_ is the thickness of the active layer, *n*_0_ is the flat band electron concentration, and *V_t_* is the thermal voltage (*kT/q*). On the other hand, *Q_i_* can also be obtained from the transfer characteristics of TFT at low *V_ds_*.

(16) $$Q_{i}\left(V_{gs}\right)=-\frac{I_{d}\left(V_{gs}\right)}{\mu \frac{W}{L}V_{ds}}$$

Note that *n*_0_ can be extracted from Equation (15) equal to Equation (16) at the condition of flat band, i.e., *Ψ_s_ =* 0 and *V_gs_* = *V_fb_*. Then, *x*_0_ at some *V_gs_* can be calculated from Equation (15) equal to Equation (16) by numerical integration.

Furthermore, based on the charge conservation relationship *Q_g_ + Q_o_ + Q_s_ =* 0, where *Q_o_* is the effective interface charge per unit area at the gate insulator, one obtains

(17) $$Q_{o}=-Q_{g}-Q_{s}$$

Thus, the interface density of states (*N_it_*) can be calculated by differentiating *Q*_0_ with respect to *Ψ_s_*

(18) $$N_{it}\left(E_{F0}+q\psi_{S}\right)=-\frac{1}{q^{2}}\frac{dQ_{0}}{d\psi_{S}}$$

where *E_F_*_0_ is the bulk Fermi level of the active layer, which is calculated from the relationship of *n*_0_ to *E_F_*_0_ :

(19) $$n_{0}=N_{C}\exp(\frac{E_{F0}-E_{C}}{kT})$$

where *N*_C_ is the effective density of states in the conduction band with a typical value of 5 × 10^18^ cm^−3^ [18].

Furthermore, based on the surface potential *Ψ_s_* given by Equation (14) and the surface charge concentration *ρ*(*Ψ_s_*) given by Equation (9), the bulk density of states can be given by [17]

(20) $$N_{bt}\left(E_{F0}+q\psi_{s}\right)=-\frac{1}{q^{2}}\frac{\rho \left(\psi_{s}+\Delta \psi_{s}\right)-\rho \left(\psi_{s}\right)}{\Delta \psi_{s}}-\frac{n_{0}}{qV_{t}}\exp\left(\frac{\psi_{s}}{V_{t}}\right)$$

The extraction procedure of DOS is described as follows. Firstly, *Ψ_s_* with respect to *V_gs_* is obtained from the *C_g_*–*V_gs_* characteristics of TFTs by Equation (14). Secondly, *x*_0_ can be calculated from the current characteristics of TFTs by using Equations (15) and (16). Thirdly, *ρ*(*Ψ_s_*), *Q_s_*, and *Q_o_* will be subsequently obtained by Equations (9), (11), and (17), respectively. Finally, the interface density of states (*N_it_*(*E*)) and the bulk density of states (*N_bt_*(*E*)) can be extracted by Equations (18) and (20), respectively. As a result, the extraction method may be easily realised step by step as seen from the above extraction procedure.

## 3. Results and Discussion

The fabrication process of the IZO TFTs is described as follows. A gate electrode of molybdenum (Mo, 200 nm) is deposited by direct current (DC) sputtering. Subsequently, a 300 nm gate insulator (SiO_2_) was deposited by plasma-enhanced chemical vapor deposition (PECVD). A 30 nm IZO active layer is deposited by using a radio frequency (RF) magnetron system with the segregated target of IZO (In_2_O_3_:ZnO = 1:1). Then, an etch stopper layer (ESL) SiO_2_ is deposited by PECVD to protect the active layer. Finally, a 200nm Mo is formed by sputtering as S/D electrodes. The channel length and width of IZO TFTs are determined by layout and patterned by the conventional lithographic techniques.

**Figure 2:**
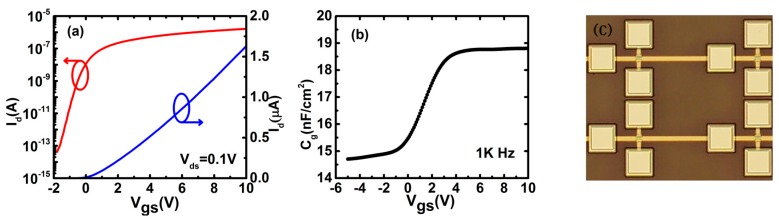
Experimental data of IZO TFTs (*W*/*L* = 20 μm/10 μm). (**a**) Transfer characteristics; (**b**) *C_g_–V_gs_* characteristics; (**c**) The micrograph of the devices.

**Figure 3:**
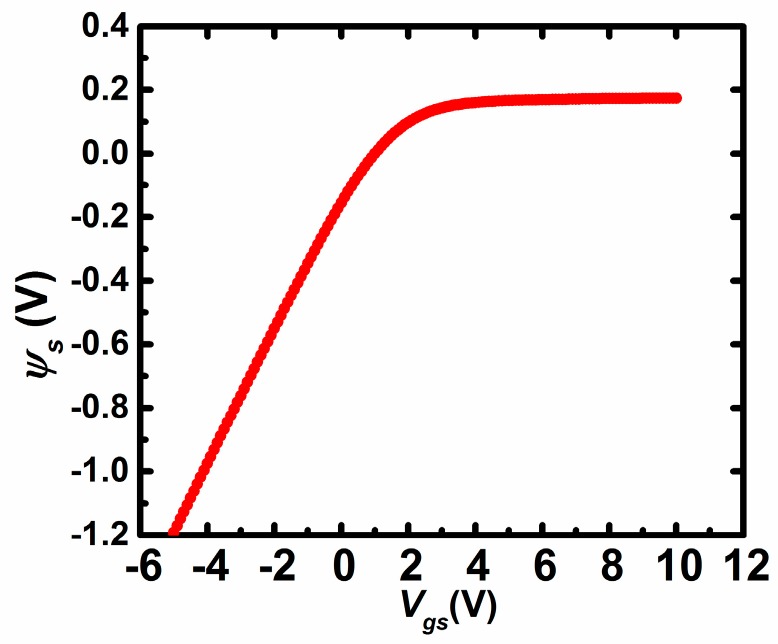
*Ψ_s_* versus *V_gs_* obtained by (14) based on the capacitance–voltage (C–V) characteristics.

The current–voltage (I–V) and C–V characteristics of TFTs are measured by using a probe station and a semiconductor parameter analyser (Agilent B1500, Agilent Technologies Inc., Santa Clara, CA, USA). The transfer characteristics are measured by scanning the gate-source voltage (*V_gs_*) from −10 V to 10 V with the step of 0.1 V at the condition of *V_ds_* = 0.1 V. The C–V characteristics are measured by superimposing the AC voltage signal (amplitude = 200 mV, frequency = 1 kHz) to DC gate bias in the condition of source and drain electrodes connected together. Note that those measurements are done at room temperature in the dark air ambient. It is thought that the DOS distribution of AOS TFTs remains unchanged at different temperatures [10]. Figure 2a shows the transfer characteristics of IZO TFTs (*W*/*L* = 20 μm/10 μm). The threshold voltage, the field-effect mobility, sub-threshold swing (SS), and on/off current ratio (I_on_/I_off_) of the pristine TFTs are extracted to be 1.8 V, 12.36 cm^2^/(V·s), 0.23 V/dec, and 1 × 10^7^, respectively. *V_fb_* is extracted as 0.6 V from the I–V characteristics following the method developed by Migliorato et al. [19]. *n*_0_ is extracted as 3.84 × 10^16^ cm^−3^ by (15) when *Ψ_s_ =* 0, i.e., *V_gs_* = *V_fb_*. Figure 2b shows the *C_g_*–*V_gs_* characteristics of the devices at the frequency of 1 kHz, which are measured with source and drain electrodes combined together. Note that the frequency of 1 kHz for the AC signal may be low enough to make the TFTs work in the quasi-static conditions. Figure 3 shows the surface potential (*Ψ_s_*) with respect to *V_gs_* from (14) based on the C–V characteristics and the value of *V_fb_*. Figure 2c shows the micrograph of IZO TFTs with *W*/*L* = 20 μm/10 μm.

**Figure 4:**
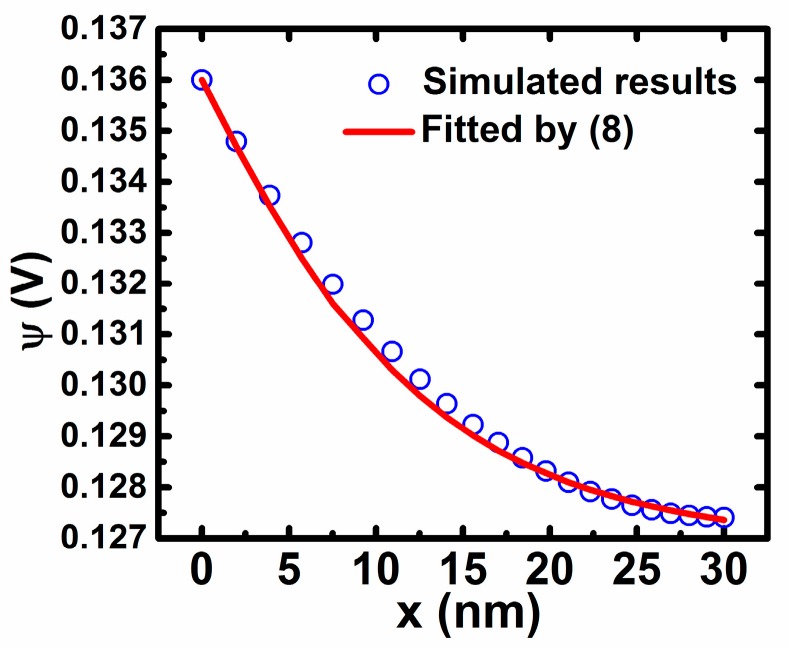
The potential distribution simulated by a 2D device simulator ATLAS.

**Figure 5:**
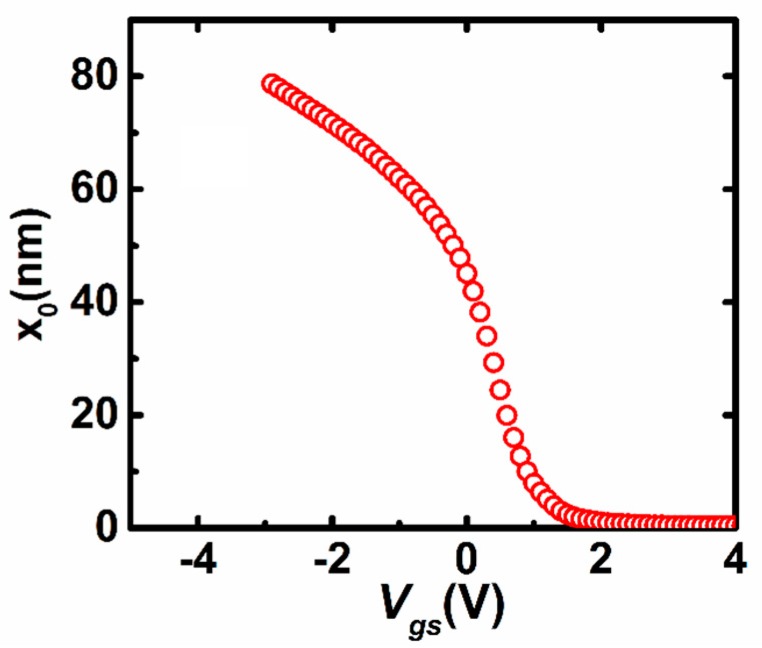
The calculated results of *x*_0_ versus *V_gs_*.

For further verification of (8), we perform the device simulation by the 2D device simulator ATLAS (Silvaco) for the IZO TFTs as described above. Figure 4 shows the distribution of the potential along the depth direction (*x*) at the conditions of *V_gs_* = 2.0 V, *V_ds_* = 0.1 V. It is found that the proposed Equation (8) can fit the simulated potential distribution well. Note that at other values of Vgs including that at the subthreshold region, the simulated potential distributions are also found to be well fitted by Equation (8) with different values of x_0_. As a result, Equation (8) may be reasonable and correct for reproducing the potential distribution of the thin active layer. Furthermore, based on the I–V characteristics and the surface potential distribution as seen in Figure 3, the value of *x*_0_ can be extracted from Equation (15) equal to Equation (16), as shown in Figure 5. It is found that the value of x_0_ tends to vary slowly when *V_gs_* is larger than the threshold voltage, because the variation of *Q_i_* changes more slowly for TFTs working in the above-threshold region.

**Figure 6:**
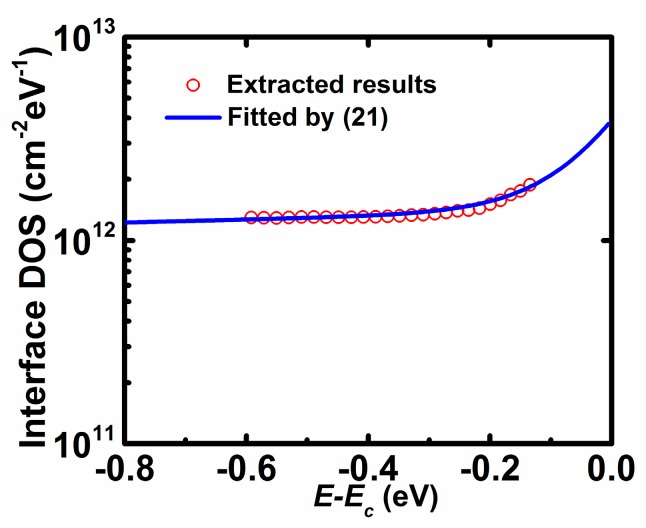
Extracted interface density of states (DOS) as a function of *E* − *E_C_* from the proposed method (symbols) and results fitted by (21) (solid lines).

Figure 6 shows the extracted interface density of states profile calculated from Equation (18). It is found that the interface DOS distribution is quite flat for the energy far from *E_C_*, while it linearly increases with the increase of energy close to *E_C_* at the exponent coordinate. Then, the interface DOS of AOS TFTs may be divided into two parts: constant deep states and exponential tail states, i.e.,

(21) $$N_{it}\left(E\right)=N_{iD}+N_{iT}\exp\left(\frac{E-E_{C}}{E_{iT}}\right)$$

where *N_iD_* is the density of deep states, *N_iT_* is the density tail states at the conduction edge, and *E_iT_* is the characteristic energy of tail states. For details, *N_iD_*/*N_iT_* is extracted by extrapolating the deep/tail states to *E* = *E*_C_, and *E_iT_* is extracted from the slope of log(*N_it_*) versus (*E* − *E*_C_) for the tail states. The extracted values are *N_iD_* = 1.3 × 10^12^ cm^−2^ eV^−1^, *N_iT_* = 2.9 × 10^12^ cm^−2^ eV^−1^, *E_iT_* = 0.08 eV. Obviously, the interface DOS distribution can be used to characterise interface quality and the reliability of AOS TFTs [20,21].

**Figure 7:**
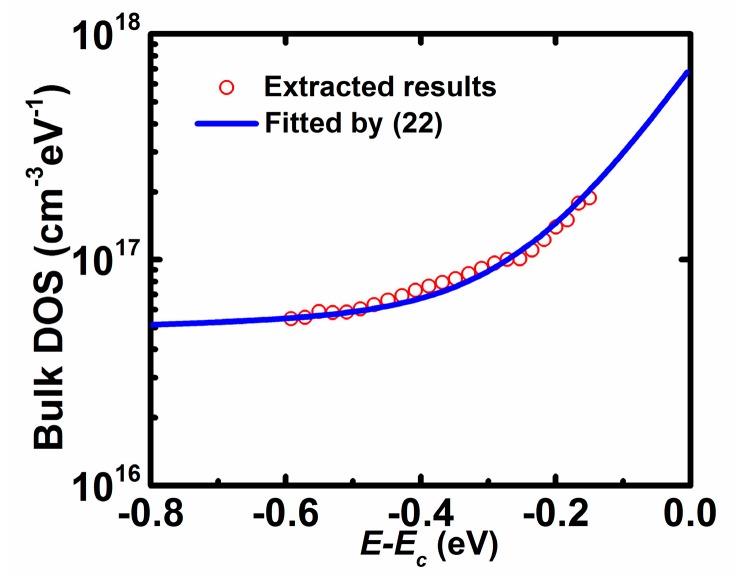
Extracted bulk DOS as a function of *E* − *E_C_* from the proposed method (symbols) and results fitted by (22) (solid lines).

Figure 7 shows the bulk density of states profile extracted from Equation (20). It is observed that the bulk DOS distribution slowly increases with the increase of the energy far from *E_C_*, while it quickly increases with the increase of energy close to *E_C_* at the exponent coordinate. It is found that the bulk DOS may be a superposition of exponential deep states and exponential tail states, i.e.,

(22) $$N_{bt}\left(E\right)=N_{bD}\exp\left(\frac{E-E_{C}}{E_{bD}}\right)+N_{bT}\exp\left(\frac{E-E_{C}}{E_{bT}}\right)$$

where *N_bD_*/*N_bT_* is the density of deep/tail states at the conduction edge and *E_bD_*/*E_bT_* is the characteristic energy of deep/tail states, of which the extraction method is similar to that of interface DOS as described above. The extracted values are *N_bD_* = 6.0 × 10^16^ cm^−3^ eV^−1^, *E_bD_* = 5.0 eV, *N_bT_* = 6.5 × 10^17^ cm^−3^ eV^−1^, and *E_bT_* = 0.10 eV. Such double exponential distribution for the bulk DOS can also be seen in other previously reported works [8,9,10,11]. For details, the deep states in AOSs may derive from oxygen deficiency, while the tail states originate from the variation of In–O–metal bonding angles [22].

**Figure 8:**
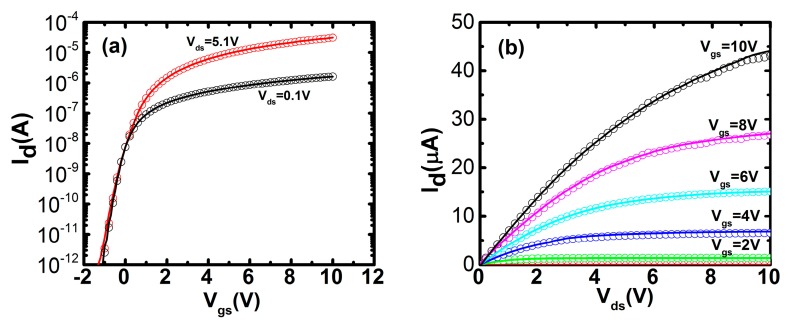
Experimental data (symbols) and simulated results (solid lines) of IZO TFTs with *W*/*L* = 20 μm/10 μm. (**a**) Transfer characteristics; (**b**) output characteristics.

The device simulation is performed by a 2D device simulator ATLAS (Silvaco, Silvaco International, Santa Clara, CA, USA) to verify the proposed method, where the extracted values of interface DOS and bulk DOS are inserted into the simulator. The other parameters of IZO TFTs in the simulation are the same as those of the fabricated IZO TFTs. As seen from Figure 8, the simulation results exhibit a good agreement with the measured transfer and output characteristics of IZO TFTs. In brief, the proposed extraction method for interface and bulk DOS is valuable for the application in metal oxide TFTs.

## 4. Conclusions

In this paper, we propose a semi-analytical extraction method for the interface DOS and the bulk DOS of IZO TFTs by using the low-frequency C–V characteristics and I–V characteristics. It is shown that the interface DOS is extracted as a superposition of constant deep states and exponential tail states with *N_iD_* = 1.3 × 10^12^ cm^−2^ eV^−1^, *N_iT_* = 2.9 × 10^12^ cm^−2^ eV^−1^, and *E_iT_* = 0.08 eV. Additionally, the bulk DOS is extracted as the superposition of exponential deep states and exponential tail states with *N_bD_* = 6.0 × 10^16^ cm^−3^ eV^−1^, *E_bD_* = 5.0 eV, *N_bT_* = 6.5 × 10^17^ cm^−3^ eV^−1^, and *E_bT_* = 0.10 eV. Furthermore, the device simulation is performed by a 2D device simulator to verify the extracted values of interface DOS and bulk DOS. It is found that there is a good agreement between simulation results and the measured transfer and output characteristics of IZO TFTs. Hence, the proposed extraction method for interface and bulk DOS may be valuable for characterising metal oxide TFTs due to the advantages of being semi-analytical and fast. Since the proposed extraction method is directly deduced from Poisson’s equation, it may also be applied in other types of TFTs, such as a-Si:H TFTs, polysilicon TFTs, or organic TFTs.

## Figures and Tables

**Figure A1:**
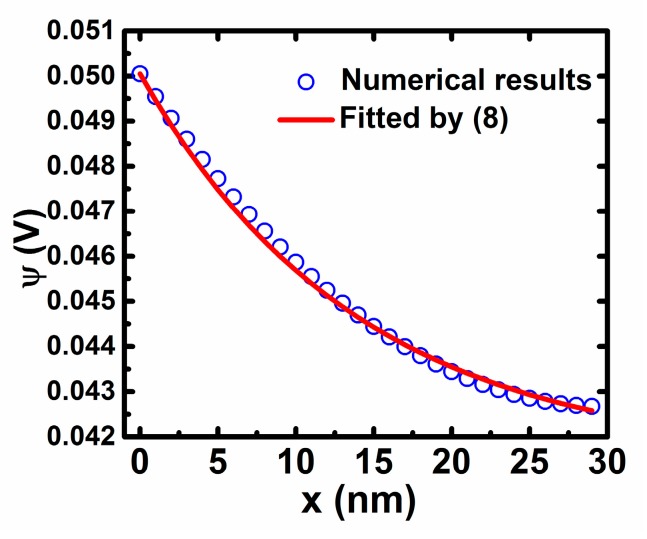
The potential distribution by numerical solution for (A5).

## Appendix A

Poisson’s equation is rewritten by

(A1) $$\frac{d^{2}\psi }{dx^{2}}=\frac{q}{\varepsilon_{s}}\left[n_{free}\left(x\right)+n_{trap}\left(x\right)\right]$$

where *n_free_*(*x*) and *n_trap_*(*x*) may be expressed as

(A2) $$n_{free}\left(x\right)=n_{0}\exp(\frac{\psi \left(x\right)}{V_{t}})$$

(A3) $$n_{trap}\left(x\right)=\int_{E_{V}}^{E_{F0}+q\psi \left(x\right)}N_{bt}\left(E\right)dE$$

Note that, Equation (A2) represents the electron concentration varying with surface band bending [7].

Substituting (22) into (A3) and integrating, we have

(A4) $$n_{trap}\left(x\right)=N_{D0}\exp\left(\frac{q\psi }{E_{bD}}\right)+N_{T0}\exp\left(\frac{q\psi }{E_{bT}}\right)-n_{t0}$$

with

$$N_{D0}=N_{bD}E_{bD}\exp\left(\frac{E_{F0}-E_{c}}{E_{bD}}\right)$$

$$N_{T0}=N_{bT}E_{bT}\exp\left(\frac{E_{F0}-E_{c}}{E_{bT}}\right)$$

$$n_{t0}=N_{bD}E_{bD}\exp\left(\frac{E_{v}-E_{c}}{E_{bD}}\right)+N_{bT}E_{bT}\exp\left(\frac{E_{v}-E_{c}}{E_{bT}}\right)$$

Then, substituting (A2) and (A4) into (A1), yields

(A5) $$\frac{d^{2}\psi }{dx^{2}}=\frac{q}{\varepsilon_{s}}\left[n_{0}\exp(\frac{\psi }{V_{t}})+N_{D0}\exp(\frac{q\psi }{E_{bD}})+N_{T0}\exp(\frac{q\psi }{E_{bT}})-n_{t0}\right]$$

Obviously, there is no analytical solution for (A5) due to its complexity. Hence, a numerical iterative solution for (A5) should be used to observe the distribution of potential.

Under the boundary conditions

(A6) $$\psi \left(0\right)=\psi_{s}\;\mathrm{and}\;{-\frac{d\psi }{dx}|}_{x=0}=E_{s}$$

where *Ψ_s_* is obtained from (14). *E_s_* is the surface electric field of the active layer, which is obtained from the relationships $\varepsilon_{ox}V_{ox}/t_{ox}=\varepsilon_{s}E_{s}$ and $V_{gs}=\varphi_{ms}+\psi_{s}+V_{ox}$, where *V_ox_* is the voltage across the oxide and *Φ_ms_* is the work function difference between the gate and active layer semiconductor.

Then

(A7) $$E_{s}=(V_{gs}-\varphi_{ms}-\psi_{s})C_{ox}/\varepsilon_{s}$$

As a result, Poisson’s equation of (A5) can be numerically solved under the boundary conditions of (A6) by using the conventional finite difference method. Figure A1 shows the numerical results of the potential along the depth direction (*x*) with the extracted values of *N_bt_*(*E*) at the condition of *V_gs_* = 1.0 V. It is shown that the numerical results of potential distribution may be reproduced well by (8). It is further found that the numerical results of potential distribution at other *V_gs_* can also be fitted well by (8) with different *x*_0_. Similarly, analytical complex exponential distributions of potential are also obtained by only taking into account either the free electrons [23] or the trapped electrons [12] in Poisson’s equation. In brief, Equation (8) may be a reasonable approximation for the potential distribution to achieve a good tradeoff between accuracy and simplicity.
